# Instructor-reported support needs and neurodevelopmental disorder diagnoses among children in Japanese judo: a cross-sectional survey across all Japanese prefectures

**DOI:** 10.3389/fspor.2026.1912597

**Published:** 2026-07-27

**Authors:** Ryosuke Ozaki, Kenji Hosokawa, Kenichi Nishimura

**Affiliations:** 1 Faculty of Sports and Budo Coaching Studies, National Institute of Fitness and Sports in Kanoya, Kanoya, Japan; 2 School of Education, Nagoya University of the Arts, Nagoya, Japan; 3 Faculty of Education, Kochi University, Kochi, Japan

**Keywords:** adapted sports, community sport, inclusive practice, instructor report, judo, neurodevelopmental disorders, youth participation

## Abstract

**Background:**

Community sport clubs serve as important participation environments for children with neurodevelopmental disorders (NDDs). Yet, nationwide data on their enrollment in specific sports remain scarce. This study estimated the proportions of participants identified by instructors as requiring additional support and instructor-known NDD diagnoses in judo clubs and programs.

**Methods:**

A cross-sectional survey covering all 47 Japanese prefectures was conducted in 2025. Instructors of 1,104 clubs and programs reported the total number of participants, the number requiring additional support, and instructor-known diagnoses of attention-deficit/hyperactivity disorder (ADHD), autism spectrum disorder (ASD), specific learning disorder (SLD), and developmental coordination disorder (DCD). Proportions and 95% confidence intervals (Wilson score method) were estimated by age category. A Cochran-Armitage trend test was used to assess linear trends across ordered age groups.

**Results:**

Overall, 1,079 of 15,360 participants (7.02%, 95% CI 6.63–7.44) were identified as requiring additional support. Proportions generally decreased with advancing age category: preschool 12.13% (10.29–14.25), elementary school 7.24% (6.66–7.88), junior high school 7.59% (6.84–8.41), high school 5.21% (4.34–6.25), and university 0.79% (0.38–1.62) (Cochran-Armitage trend test: Z = –8.64, *p* < 0.001). Instructor-known diagnoses were reported for 1.57% (ADHD), 0.68% (ASD), 0.70% (SLD), and 0.09% (DCD) of participants. Diagnostic categories were not mutually exclusive. The summed proportion of instructor-known diagnostic reports (3.04%) therefore represents an upper bound on the proportion of participants with any known diagnosis. Even this upper bound remained well below the instructor-identified support-need proportion (7.02%). This gap indicates that support needs in community judo extend beyond disclosed diagnoses.

**Conclusions:**

Participants requiring additional support are present in Japanese judo clubs and programs across prefectures. Crucially, diagnosis-independent, and age- and setting-sensitive accommodation in community sport is important. Finally, the 7.02% figure should be interpreted as a context-specific indicator of the support demand instructors perceive rather than as a clinical prevalence estimate.

## 1 Introduction

Neurodevelopmental disorders (NDDs), including autism spectrum disorder (ASD), attention-deficit/hyperactivity disorder (ADHD), intellectual developmental disorder, and specific learning disorder (SLD), arise during central-nervous-system development. They are associated with difficulties in attention regulation, impulse control, social communication, and behavioral flexibility (1). Children with NDDs engage in lower habitual physical activity than their typically developing peers (2). Further, psychiatric comorbidities such as depression are common across the life course (3, 4). Therefore, sport and exercise interventions are being increasingly examined as non-pharmacological strategies for supporting physical, cognitive, and mental-health outcomes in this population (5).

Within this broader agenda, two parallel trends frame the present study. First, sport-participation research has begun to differentiate trajectories by sport type and social context: Australian youth with autism exhibit lower participation in team sports than those without autism (6), and children and adolescents with autism in Europe favor individual sports over team sports (7). Second, intervention research has highlighted martial arts, particularly judo, as a structured movement form that may benefit executive function and behavior in children with ADHD and ASD (8–11). However, the field has largely been built on either large epidemiological samples without sport-specific resolution or small intervention studies without population-scale anchoring. To the best of our knowledge, large-scale data on who actually participates in a specific sport, in a specific country, and across the developmental span have been absent.

This gap is particularly conspicuous in Asia, with limited evidence on the sport participation of children with NDDs. Japan provides a particularly relevant context for addressing this gap: It is the birthplace of judo, and judo instruction is delivered nationwide through community dojos and school-affiliated programs that span preschool through university. Nevertheless, the most recent national survey of community youth sports in Japan did not stratify by NDDs or specific sports, such as judo, soccer, or baseball (12). Meanwhile, practice-based reports on judo as a developmental support modality have accumulated (13–18). For instance, a single prefecture-level survey suggested that community judo clubs already serve children with NDD-related characteristics (19). Yet, no large-scale instructor-reported survey has clarified how widely such support needs are encountered across Japanese judo settings. This absence is consequential: Without nationwide data, policy discussions about inclusive coach education, club-level accommodation, and disclosure norms in Japanese community sport remain limited by the lack of an empirical foundation.

We approached the problem through a participation-oriented lens informed by the International Classification of Functioning, Disability, and Health (ICF) framework. This framework treats sport participation as the dynamic interaction between individual functioning, the activity, and contextual factors (20). From this perspective, an instructor's recognition that a participant requires additional support is itself an environmental signal: It reflects both the individual's functioning, and the demands and supports embedded in the community judo context. Therefore, counting these instructor-identified support needs at scale offers a different, and complementary, window on inclusion compared with diagnosis-based prevalence estimates.

Building on this framing, this study makes three contributions to the literature. First, we provide nationwide instructor-reported evidence on the enrollment of participants requiring additional support in a traditional Asian martial art, addressing a long-standing geographic and disciplinary gap in adapted sport research. Second, we empirically examine the extent to which instructor-identified support needs extend beyond disclosed diagnostic information. Specifically, we highlight the limitations of exclusively relying on diagnostic labels in community sport settings and uncover the practical question of how non-disclosed support needs are to be addressed. Third, we characterize the age-category pattern of observable support needs across childhood and adolescence in judo, generating specific hypotheses about retention, accommodation, and disclosure; future longitudinal and intervention work can test these. To address these aims, we have conducted a nationwide cross-sectional survey of community judo clubs in Japan (*N* = 1,104 clubs; 15,360 participants).

## 2 Materials and methods

### 2.1 Study design and setting

A cross-sectional survey covering all 47 prefectures of Japan was conducted to examine the enrollment of participants requiring additional support in judo clubs and programs. In this study, the term “judo clubs” was broadly used to include community dojos and school-affiliated judo programs at junior high schools, high schools, and universities. Data were collected using an online questionnaire distributed in 2025. Reporting follows the Strengthening the Reporting of Observational Studies in Epidemiology (STROBE) statement for cross-sectional studies.

### 2.2 Participants and recruitment

Invitations to participate in the survey were distributed through the All Japan Judo Federation's online portal, Judo-Member, to individuals holding instructor licenses whose email addresses were registered in the system. An invitation email that included an explanation of the survey and a link to the online questionnaire was sent on September 16, 2025, and responses were accepted until October 31, 2025. To minimize duplicate reporting, the survey instructions specified that only one response should be submitted per judo club. However, the federation could not provide information on the total number of invitations distributed by email. Therefore, the exact number of eligible recipients, and thus, the response rate could not be determined.

### 2.3 Definition of participants requiring additional support

Participants requiring additional support were defined using the following procedure. First, three experts reviewed the characteristics of NDDs based on the Diagnostic and Statistical Manual of Mental Disorders, Fifth Edition, Text Revision (DSM-5-TR) (1). The expert panel comprised one university faculty member who specialized in judo competition and held a fifth-dan rank; one university faculty member who specialized in special-needs education, and was both a Certified Public Psychologist and judo instructor; and one specialist in exercise support for children with disabilities. Over three iterative meetings, the panel discussed how NDD-related characteristics may manifest in judo club settings. Consensus was achieved through structured review and revision of candidate items. The panel derived five observable behavioral indicators from core NDD symptom domains described in the DSM-5-TR, each operationalized for the judo practice context. This development process—grounding each indicator in DSM-5-TR symptom domains and refining the wording through iterative multidisciplinary consensus—was intended to support the content relevance of the indicators. However, the questionnaire did not undergo formal pilot testing, and the reliability and validity of the indicators were not formally assessed before deployment.

The first indicator, difficulty in understanding the content of instructions, reflects deficits in social communication and cognitive processing commonly associated with ASD and intellectual developmental disorder (1). Examples of marked difficulty include persistently being unable to understand technique instructions even after repeated individual explanation; consistently failing to translate multi-step verbal sequences (e.g., the steps of a throwing technique) into physical movement despite one-on-one guidance; and showing no behavioral response to verbal instructions that peers readily follow. The second indicator, inability to sustain attention during practice, captures the inattentive symptom domain of ADHD and is frequently observed in ASD (1). Examples of marked inattention include being unable to maintain focus for even brief segments of practice without individual prompting; repeatedly losing attention throughout a single drilling session despite re-engagement efforts; and being so readily distracted by ambient stimuli on the tatami (e.g., sounds, lighting changes, or the movements of other practitioners) that sustained participation in any practice activity is substantially impaired. The third indicator, inability to engage in practice together with peers, reflects difficulties in social interaction that are primary features of ASD and may be further exacerbated by impulsivity in ADHD (1). Examples include refusing or being unable to participate in partner-based drills such as *uchi-komi* or *randori*; difficulty adapting to a partner's pace during cooperative practice; and withdrawal from or disproportionate reactions to physical contact. The fourth indicator, engaging in behaviors that interfere with the progress of practice, operationalizes the hyperactive-impulsive symptom domain of ADHD and the behavioral inflexibility associated with ASD (1). Examples include leaving the tatami without permission during practice; producing disruptive vocalizations or actions that interrupt group instruction; and persistent repetition of a specific movement or refusal to transition to new activities. The fifth indicator, marked clumsiness in physical movement and motor control compared with same-age peers, reflects the motor coordination difficulties characteristic of developmental coordination disorder (DCD), which frequently co-occurs with ADHD and ASD (1); concrete examples of this indicator provided to instructors in the questionnaire included inability to perform a forward roll, inability to execute mae-mawari ukemi (forward breakfall), and difficulty learning throwing and grappling techniques.

The five indicators were presented to instructors as the operational criteria defining a participant requiring additional support. For each age category (preschool, elementary school, junior high school, high school, and university), instructors reported, in a single count, the number of currently enrolled participants who showed one or more of these five indicators during judo practice. Because the count was elicited as one figure per age category rather than separately for each indicator, a participant exhibiting multiple indicators was counted only once; the numerator therefore reflects distinct participants and is not a sum across indicators. This approach was adopted to capture support-relevant functioning beyond what formal diagnostic disclosure alone can convey, given that many participants in community sport settings may present with NDD-related characteristics without a known diagnosis. Importantly, the five indicators are not specific to NDDs: Similar difficulties may also arise from factors unrelated to NDDs, such as typical developmental variability, limited experience with judo, or transient situational factors. The implications of this non-specificity are addressed in the Limitations section.

### 2.4 Survey instrument and measures

The survey was administered through an online FormRun questionnaire platform. The questionnaire included items on basic club characteristics, the number of participants requiring additional support, and NDD diagnoses known to instructors. Basic club characteristics included the prefecture in which the club was located and the participants' age categories. Participants' age was categorized based on whether they belonged to preschools, elementary schools, junior high schools, high schools, or universities.

Respondents in each age category were asked to report the total number of club participants and the number of participants requiring additional support. They were also asked to report the number of judo-club participants who had been diagnosed with NDDs, specifically ADHD, ASD, SLD, or DCD. These four categories were selected because they are the NDDs most likely to be relevant to, and observable in, judo practice settings, and because limiting the list to four reduced respondent burden. Other NDDs, such as intellectual developmental disorder, were not asked as separate diagnostic items. These data reflected diagnoses known to instructors through information disclosed by participants, parents or guardians, school personnel, or other relevant persons, and did not represent diagnoses made by the respondents themselves. The questionnaire was designed to capture the overall number of participants requiring additional support and distribution of NDD diagnoses known to instructors across age categories in community judo settings. This survey was designed to capture participant characteristics as perceived by judo instructors in practice settings, and did not involve medical or clinical judgment. Accordingly, the reported results should not be interpreted as indicating the presence or absence of NDDs in any individual participant.

### 2.5 Data processing and statistical analysis

Before analysis, the collected data were cleaned in R according to pre-specified rules established by the research team. Missing values were treated as not available (NA); where appropriate, missing numeric entries were recoded as 0. Responses containing numeric information in text form (e.g., “1 person” or “2 people”) were converted to numeric values, and leading zeros were removed. Responses clearly indicating no applicable cases (e.g., “none” or “no applicable cases”) were also recoded as 0.

Several apparent input errors or coding inconsistencies were corrected according to predefined rules. These corrections were made only when the research team judged that the intended numeric value could be reasonably inferred from the response pattern and relevant age-category context. For example, values such as 818, 200, 231, and 212 were interpreted as apparent input errors, and recoded as 18, 20, 31, and 12, respectively. Range-type entries such as “30 to 40” and “40–50” were recoded as 30 and 40, respectively, using the lower bound of the reported range to avoid overestimating the number of participants.

Nonstandard responses were processed according to predefined rules. When respondents provided narrative answers to fields that requested numeric values or responses could not be interpreted as valid numeric values, those entries were recoded as 1 to retain the cases for aggregation. Recoding these entries as 1, rather than treating them as missing, was considered preferable for two reasons: First, a substantive response had been entered in these fields, indicating the presence of at least one applicable case rather than a skipped item; second, treating these entries as missing would have discarded otherwise valid information provided by the same clubs. Assigning the minimum positive count of 1 therefore retained these cases while minimizing the risk of overestimation. Recoding these entries as 0 or treating them as missing (NA) instead of 1 did not materially alter the overall proportion of participants requiring additional support (range across scenarios: 6.97%–7.04%), showing that the handling of these nonstandard responses did not drive the main findings. The total number of recoded nonstandard responses was 40, representing 0.26% of the analyzed data.

After data cleaning, descriptive statistics were used to summarize the number of responding clubs, participants, participants requiring additional support, and NDD diagnoses known to instructors. The proportion of participants requiring additional support in each category was calculated by dividing the number of participants requiring additional support by the total number of participants. The diagnostic frequencies for ADHD, ASD, SLD, and DCD were also summarized, with percentages calculated using the total number of participants across the responding clubs as the denominator. Next, 95% confidence intervals (CIs) were calculated for all proportions using the Wilson score method. The Cochran-Armitage trend test was used to assess linear trends in proportions across ordered age categories. Chi-square tests were used to evaluate overall homogeneity across the five age categories and pairwise differences between adjacent categories; pairwise comparisons were not adjusted for multiple testing. Two-sided *p* < 0.05 was considered statistically significant. Because participant-level records were aggregated at the club and age-category levels, inferential tests were exploratory in nature and the proportions with their 95% confidence intervals (rather than the *p*-values) were treated as the primary basis for interpretation. The results should be cautiously interpreted because clustering within clubs was not modeled, which may have underestimated the standard errors. All analyses were performed in R (R Foundation for Statistical Computing, Vienna, Austria).

## 3 Results

### 3.1 Response volume and sample coverage

In total, 1,104 responses were collected from judo clubs in all 47 prefectures of Japan (Table 1). According to the All Japan Judo Federation, 7,128 judo clubs were registered nationwide as of February 25, 2025 (21). Using this figure as a reference denominator, the 1,104 responses correspond to approximately 15.5% of registered clubs. Because the actual number of distributed survey invitations was unavailable and submission of only one response per club could not be strictly verified, this figure reflects the proportion of nationally registered clubs represented in the dataset rather than a formal response rate, and should not be interpreted as an indicator of survey participation.

**Table 1:** Number of responses by prefecture.

Prefecture	n	Prefecture	n	Prefecture	n
Hokkaido	47	Ishikawa	16	Okayama	19
Aomori	19	Fukui	13	Hiroshima	16
Iwate	28	Yamanashi	8	Yamaguchi	12
Miyagi	23	Nagano	19	Tokushima	15
Akita	23	Gifu	16	Kagawa	13
Yamagata	17	Shizuoka	24	Ehime	10
Fukushima	30	Aichi	48	Kochi	13
Ibaraki	31	Mie	25	Fukuoka	40
Tochigi	22	Shiga	14	Saga	11
Gunma	21	Kyoto	13	Nagasaki	22
Saitama	50	Osaka	56	Kumamoto	21
Chiba	40	Hyogo	53	Oita	16
Tokyo	63	Nara	8	Miyazaki	13
Kanagawa	40	Wakayama	17	Kagoshima	26
Niigata	26	Tottori	10	Okinawa	2
Toyama	19	Shimane	16	Total	1,104

n, number of responses. Responses were collected from all 47 prefectures of Japan (total *N* = 1,104).

### 3.2 Characteristics of responding clubs

Our geographical coverage extended to all 47 prefectures, although the number of responses varied across prefectures. Table 2 shows the number of responding clubs according to the participants' age category. Because many clubs included participants from multiple age groups, a single club could be counted in more than one age category. Some response profiles indicated school-affiliated rather than community clubs (junior high school only, *n* = 204; high school only, *n* = 200; junior high school and high school only, *n* = 25; and university only, *n* = 42). Identifying these school-affiliated profiles is relevant to the interpretation of the findings because such programs may differ from community dojos in competitive selection, disclosure norms, and instructor familiarity with participants (see Section 4.2).

**Table 2:** Number of responding judo clubs by participants' age category.

Age category	Number of clubs
Preschool	296
Elementary school	490
Junior high school	574
High school	334
University	101

Clubs could represent more than one age category because many included participants from multiple age groups. Specific counts are reported in the aggregated dataset available in the public repository.

### 3.3 Enrollment of participants requiring additional support by age category

Overall, 1,079 of 15,360 participants (7.02%, 95% CI 6.63–7.44) were identified by their instructors as requiring additional support. The proportion differed across age categories (Table 3): 12.13% (95% CI 10.29–14.25) for preschool participants, 7.24% (6.66–7.88) for elementary-school participants, 7.59% (6.84–8.41) for junior-high-school participants, 5.21% (4.34–6.25) for high-school participants, and 0.79% (0.38–1.62) for university participants.

**Table 3:** Number of participants, number of participants requiring additional support, and proportion (with 95% confidence intervals) by age category.

Age category	Total participants (n)	Requiring additional support (n)	Proportion (%)	95% CI (Wilson)
Preschool	1,047	127	12.13	10.29–14.25
Elementary school	6,972	505	7.24	6.66–7.88
Junior high school	4,361	331	7.59	6.84–8.41
High school	2,092	109	5.21	4.34–6.25
University	888	7	0.79	0.38–1.62
Overall	15,360	1,079	7.02	6.63–7.44

Wilson score 95% confidence intervals (CI). Cochran-Armitage trend test across the five age categories: Z = –8.64, *p* < 0.001. Chi-square homogeneity test: *χ*^2^ = 107.85, df = 4, *p* < 0.001.

A test of homogeneity across the five age categories indicated significant differences in proportion (*χ*^2^ = 107.85, df = 4, *p* < 0.001). Next, a Cochran-Armitage trend test indicated a statistically significant decreasing trend with advancing age category (Z = –8.64, *p* < 0.001). Pairwise comparisons of adjacent age categories (uncorrected for multiple testing) revealed significant declines between preschool and elementary school (*χ*^2^ = 29.94, *p* < 0.001), junior high and high school (*χ*^2^ = 12.60, *p* < 0.001), and high school and university (*χ*^2^ = 32.58, *p* < 0.001); the difference between elementary and junior high school did not reach statistical significance (*χ*^2^ = 0.47, *p* = 0.49). Because participants were nested within clubs and this clustering was not modeled, these *p*-values may understate uncertainty. Moreover, age category was partially confounded with club type (see Section 3.2); the age-related differences should therefore be interpreted cautiously (see Section 4.2).

### 3.4 Instructor-known diagnoses of neurodevelopmental disorders

Table 4 shows the number and proportion of instructor-known NDD diagnostic reports. ADHD was the most frequently reported diagnosis (*n* = 241; 1.57%, 95% CI 1.38–1.78), followed by SLD (*n* = 108; 0.70%, 0.58–0.85), ASD (*n* = 104; 0.68%, 0.56–0.82), and DCD (*n* = 14; 0.09%, 0.05–0.15). Because the diagnostic categories were not mutually exclusive, the summed figure of 3.04% represents an upper bound on the proportion of distinct participants with any known diagnosis. Even this upper bound remained substantially lower than the overall instructor-identified support-need proportion (7.02%), indicating that support needs in community judo settings extend beyond cases in which diagnostic information has been disclosed to instructors.

**Table 4:** Number and proportion (with 95% confidence intervals) of instructor-known neurodevelopmental disorder diagnostic reports.

Diagnosis	n	Proportion (%)	95% CI (Wilson)
ADHD	241	1.57	1.38–1.78
ASD	104	0.68	0.56–0.82
SLD	108	0.70	0.58–0.85
DCD	14	0.09	0.05–0.15

Wilson score 95% confidence intervals (CI). Denominator is the total number of participants across the 1,104 responding clubs (N = 15,360). Diagnoses reflect information reported to instructors. ADHD, attention-deficit/hyperactivity disorder; ASD, autism spectrum disorder; SLD, specific learning disorder; DCD, developmental coordination disorder. The diagnostic categories were not mutually exclusive.

## 4 Discussion

### 4.1 Nationwide presence of participants requiring additional support in judo clubs and programs

Drawing on cross-sectional data from 1,104 community judo clubs across all Japanese prefectures, we estimated that 1,079 of 15,360 participants (7.02%, 95% CI 6.63–7.44) were identified by instructors as requiring additional support during judo instruction. Crucially, instructors across all 47 prefectures reported encountering participants requiring additional support, suggesting that such needs were reported beyond a small number of isolated regions. This finding addresses a gap identified in prior work: While inclusion and support in community sport have been conceptually articulated (22, 23), how often instructors actually encounter participants requiring support in a specific sport at the national scale has been largely unclear. Compared with intervention studies demonstrating benefits of adapted judo programs (8–11), our data help situate the intervention literature within the broader reality of judo practice reported by instructors across Japan: A meaningful number of children for whom such adaptations are relevant are already enrolled.

Rather than being an epidemiological prevalence rate, the 7.02% estimate is best interpreted as a context-specific signal of the support demand instructors face. Notably, it converges in magnitude with, but is conceptually distinct from, the general-population estimates of children with developmental support needs in Japan. For instance, a 2022 Ministry of Education, Culture, Sports, Science and Technology (MEXT) nationwide survey estimated that approximately 8.8% of children in regular classrooms exhibit potential learning or behavioral difficulties suggestive of developmental support needs (24). However, this comparison warrants considerable caution: The MEXT survey used standardized teacher reports in compulsory education, whereas we used non-validated, instructor-defined criteria in a voluntary sport setting, and the two surveys differ in age coverage, respondents, and sampling procedures. Accordingly, the similar order of magnitude should not be read as evidence of equivalence; at most, it suggests that the level of support needs reported by judo instructors is not negligible. This is meaningful because community sport has often been assumed to underrepresent children with NDDs relative to mainstream education (12, 25). Meanwhile, our data suggest that, at least among responding clubs, support needs are not rare in judo settings.

These findings suggest that support needs in judo settings are not confined to a small number of exceptional cases but constitute an issue broadly shared across instructional settings, highlighting the need to systematically document the support strategies judo instructors already use and to connect this knowledge to future instructor training and support systems.

### 4.2 Age-category differences in enrollment proportions and their implications

The proportion of participants recognized as requiring additional support generally decreased with advancing age category, with a statistically significant decreasing trend. Declines were concentrated at the boundaries between developmental and educational stages rather than continuously across childhood (see results, Section 3.3).

However, this statistically significant age-category gradient cannot be interpreted directly as evidence of dropout or developmental change because the design is cross-sectional and individual continuation was not tracked. In addition, because clustering within clubs was not modeled, the strength of the statistical evidence for this gradient may be overstated. Moreover, the age categories systematically differ in club type: Sampled university and high-school clubs included substantial proportions of school-affiliated, competitively oriented programs (university only, *n* = 42; high school only, *n* = 200). These may differ from community dojos in selection pressure, disclosure norms, and instructor familiarity with participants. The sharp decline at the university level in particular (0.79%) likely reflects competitive selection into university judo programs as much as any developmental trend. Because club type was confounded with age category and could not be disentangled in the present aggregated data, the age gradient should be regarded as partly attributable to differences in club composition. Therefore, a sensitivity analysis restricted to community dojos (excluding school-affiliated programs) is warranted in future work to separate competitive selection from developmental and disclosure-related factors. Essentially, at least three non-mutually-exclusive explanations are plausible.

First, age-related changes in competitive environments may be relevant. As children grow older, judo generally places greater demands on rule-governed behavior, sustained attention, self-regulation, interpersonal adjustment, and consistent motor performance. The characteristics associated with NDDs span multiple domains, including attentional control, impulsivity, social communication, and sensory processing (1). Therefore, difficulties in judo practice may become more apparent with age, making continued participation challenging. Additionally, the participation of children, including those with NDDs, in physical activities may be hindered by barriers such as motivation, self-efficacy, interpersonal relationships, safety considerations, and staff training (26). Meanwhile, their inclusion in sports clubs depends on adaptive coaching, appropriate activity design, and the availability of external support (22). Thus, the observed age-related differences may not reflect age itself but rather how support systems and environmental adjustments keep pace with increasing competitive demands.

Second, continued participation in judo and developmental change may have reduced the visibility of difficulties over time. Randomized controlled trials in children with ADHD have shown that judo training may have beneficial effects on executive functions, such as working memory (8, 9). Adapting judo programs to children with ASD has been associated with behavioral improvements (10, 11). Therefore, the age-related differences observed here may reflect not only the discontinuation of some children's participation but also the possibility that continued judo practice and developmental maturation reduce the likelihood that some children are recognized as requiring additional support. However, the present data do not distinguish these possibilities.

Third, how diagnostic and trait-related information is shared may influence the interpretation of age-related differences. A scoping review of neurodiversity in sport has suggested that inadequate environmental adjustments, limited understanding from others, and circumstances in which individuals find it difficult to request support or conceal their characteristics may shape participation experiences (27). Under such conditions, instructors may not recognize participants who require support and remain enrolled. This may be particularly relevant from high school onward, when both adolescents and their parents may become more cautious about disclosing diagnoses or related characteristics. Therefore, our observed decline may reflect not only actual enrollment patterns but also reduced detectability.

Thus, a single factor may not explain the age-related differences identified here; rather, these differences likely reflect the combined influence of the three sets of factors outlined above. Therefore, support provided in judo settings should be tailored to age category, club type, competitive context, and disclosure-related needs, and designed to promote continued participation.

### 4.3 Beyond diagnostic labels: support needs identified in practice

Support needs cannot be fully captured by diagnostic labels alone. Here, diagnoses known to instructors were reported for 1.57%, 0.68%, 0.70%, and 0.09% of participants for ADHD, ASD, SLD, and DCD, respectively. Thus, participants diagnosed with NDDs do participate in judo clubs. Further, this diagnostic information is shared with instructors in at least some cases. In Japan, barriers to sports participation among individuals with NDDs include the lack of acceptability or receptive settings, individuals' refusal to participate, and more passive selection of sports (25). Herein, our findings suggest that some judo clubs function as actual participation sites.

Meanwhile, the diagnosed-participant counts were limited to cases in which the diagnosis had been disclosed to instructors by parents, participants themselves, or others; undiagnosed participants and undisclosed diagnoses were therefore not captured. As shown in Section 3.4, even the upper-bound estimate of participants with any known diagnosis (≤ 3.04%) fell well below the 7.02% identified as requiring support. This contrast indicates that instructor-identified support needs extend beyond disclosed diagnostic information. Therefore, support in judo clubs should not be based solely on the presence or absence of a diagnosis but should also respond to observed difficulties in actual practice, with accommodations and support flexibly organized in response to those needs.

### 4.4 Practical and research implications

The findings suggest four possible directions for inclusive coaching, policy, and research in Japanese community judo. Given the cross-sectional and instructor-reported nature of the data, these directions should be read as hypothesis-generating rather than prescriptive.

#### 4.4.1 Inclusive coach education as a baseline competency

Given that approximately 1 in 14 participants in the responding clubs was identified as requiring additional support, instructor licensing or renewal programs could consider incorporating brief standardized modules on neurodevelopmental support, covering the recognition of NDD-related behavioral patterns in dojo settings, principles of reasonable accommodation, and case-based problem solving.

#### 4.4.2 Stage-graded accommodation rather than one-size-fits-all inclusion

Because support-need recognition was highest at preschool age and decreased through high school, accommodation strategies may need to be differentiated across age groups. Preschool clubs may prioritize sensory regulation and behavioral predictability, elementary and junior-high clubs may emphasize peer-mediated cooperative practice, and high-school and university clubs may focus on self-advocacy and disclosure decisions.

#### 4.4.3 Diagnosis-independent accommodation policies

Given that instructor-identified support needs exceeded the disclosed diagnostic information (Section 4.3), clubs may be advised not to treat formal diagnostic information as a precondition for considering accommodation. Policy templates that frame accommodation as responsive to observed difficulty, rather than to documented diagnosis, may lower the disclosure burden on families.

#### 4.4.4 Longitudinal and mixed-methods follow-up

To distinguish among the three explanations of the age gradient (competitive demands, developmental progression, and disclosure dynamics), future work should follow individual participants over time and triangulate instructor reports with participant and parent perspectives. Ideally, this should be performed using validated instruments such as adapted versions of the Strengths and Difficulties Questionnaire, alongside dropout tracking.

Finally, the findings provide initial, cross-sectional support for reconceptualizing community sport as a routine site of support for children with NDDs, rather than merely a specialized one. The conceptual shift implied is from “adapted programs serving identified populations” to “adaptive instruction serving an inherently heterogeneous participation pool.”

### 4.5 Limitations

In terms of sampling, an accurate response rate could not be calculated because the actual number of survey invitations distributed was unavailable. Further, duplicate responses could not be rigorously verified despite instructions to submit one response per club. Participation was voluntary; as such, clubs with greater awareness of or interest in inclusive practice may have been more likely to respond, potentially inflating the reported proportions. Because the direction and magnitude of this potential self-selection cannot be quantified from the available data, the representativeness of the responding clubs remains uncertain, and the reported proportions should be generalized to Japanese judo settings as a whole with caution.

Next, on measurement, participants requiring additional support were identified through instructor observation rather than standardized assessment by physicians, psychologists, or other professionals. Furthermore, the inter-rater reliability of instructor judgments was not assessed. As such, the same observable behaviors may be perceived differently across instructors and contexts. Moreover, the five behavioral indicators were derived through expert consensus but were not psychometrically validated. Accordingly, the support-need proportion should be understood as an instructor-perceived indicator of support demand rather than a validated measure of NDD-related functioning. Relatedly, because the indicators are not specific to NDDs, some participants may have been identified owing to difficulties arising from factors unrelated to NDDs, whereas others with genuine support needs may not have been recognized; the extent of such misclassification cannot be estimated from the present data. Instructor-known diagnoses were limited to information disclosed to instructors and may exclude undiagnosed participants and those whose diagnostic status had not been shared. Because diagnostic categories were not mutually exclusive, the summed proportion of diagnostic reports (3.04%) cannot be interpreted as the proportion of participants with any NDD diagnosis.

Finally, regarding analysis and design, this study did not stratify by club size, competitive level, training frequency, regional characteristics, or club type (community dojos versus school-affiliated programs). These may systematically differ, and may have contributed to the observed age-category gradient. Some nonstandard responses were recoded during data cleaning. While sensitivity analyses showed that alternative recoding strategies did not materially alter the main estimates (range: 6.97%–7.04%), a slight influence cannot be entirely excluded. The cluster structure of the data (participants nested within clubs) was not accounted for in the statistical analysis, which may have led to underestimation of standard errors. As this was a cross-sectional study, whether the observed age-category differences reflected continued participation, withdrawal, support effects, competitive selection, or reduced detectability could not be determined. Future qualitative and longitudinal research should examine these questions, including actual support practices in judo clubs, the effects of instructor training, and the perspectives of participants and their families.

## 5 Conclusion

This cross-sectional instructor survey covering all 47 Japanese prefectures revealed that 7.02% (95% CI 6.63–7.44) of participants in the responding judo clubs and programs were identified as requiring additional support. A statistically significant age-category gradient was observed (Z = –8.64, *p* < 0.001). However, this pattern may reflect multiple interacting factors including competitive selection, club type, disclosure practices, and developmental change. The summed proportion of instructor-known diagnostic reports (3.04%), representing an upper bound on the proportion of participants with any known diagnosis, was lower than the overall support-need proportion. Thus, disclosed diagnostic information alone may not fully capture the support needs in judo settings. As such, accommodation approaches based on observed difficulties in practice should be considered. Finally, future longitudinal and mixed-methods studies should clarify the factors underlying continued participation and support needs.

## Data Availability

The original contributions presented in the study are included in the article/Supplementary Material, further inquiries can be directed to the corresponding author.
